# Endometriosis-Associated Massive Ascites in an Asian Woman: A Case Report of a Rare Clinical Entity

**DOI:** 10.1155/2020/8879643

**Published:** 2020-08-04

**Authors:** Nuntasiri Eamudomkarn, Naratassapol Likitdee, Pilaiwan Kleebkaow, Chumnan Kietpeerakool

**Affiliations:** Department of Obstetrics and Gynaecology, Faculty of Medicine, Khon Kaen University, Khon Kaen, Thailand

## Abstract

Massive ascites as a presentation of endometriosis is a rare clinical entity that is most commonly seen in black nulliparous females. Herein, we describe a case of a 32-year-old multiparous Thai woman who presented with a two-year history of abdominal distension. Computerized tomography of the abdominopelvic region showed an infiltrative enhancing lesion involving the cul-de-sac and perirectal region with massive loculated ascites, suggesting carcinomatosis peritonei. Abdominal paracentesis was performed to yield fluid samples for evaluation, which revealed no malignant cells, and polymerase chain reaction (PCR) was negative for tuberculosis. The patient underwent exploratory laparotomy which revealed a large amount of serosanguinous ascites, thickened matted bowel loops, and necrotic debris covering the entire surface of the peritoneum and visceral organs. The surgical procedures included drainage of 6.5 liters of ascites, lysis adhesion, biopsy of the peritoneum, and right salpingo-oophorectomy. Histologic examination revealed benign endometrial glands with stroma at the peritoneum tissue and broad ligament. Other causes of ascites were excluded. The ascites responded to drainage and hormonal suppression. A final diagnosis of endometriosis was made based on these findings. Endometriosis should therefore be considered in differential diagnosis in women of childbearing age who present with ascites.

## 1. Introduction

Endometriosis is a benign, estrogen-dependent disease characterized by endometrial tissue outside the uterine cavity [1, 2]. Endometrial implantation can be in either the pelvic or extrapelvic region with the former being more prevalent [3, 4]. Clinical presentations vary by endometrial implantation site. Typical symptoms include chronic pelvic pain, dysmenorrhea, dyspareunia, and infertility [5].

The occurrence of ascites secondary to endometriosis is rarely encountered. It appears to occur more often in black nulliparous females [6]. Therefore, the accumulation of case reports regarding this rare clinical entity, particularly cases with distinct clinical backgrounds, is necessary in order to investigate its clinical course. Herein, we report a case of massive ascites associated with endometriosis in a reproductive-aged Thai woman who presented with massive ascites, significant weight loss, and peritoneal lesions mimicking malignancy. For literature review, we searched PubMed using the following key words; endometriosis, ascites, and pathogenesis.

## 2. Case Report

A 32-year-old woman, G1P1001, presented with two years of increasing abdominal distension. She had also experienced poor appetite and weight loss. Her menstrual period was regular, and she had neither abnormal vaginal bleeding nor pelvic pain.

Six years prior to this visit, she experienced chronic pelvic pain and had been treated with injectable progestin for clinical suspicion of pelvic endometriosis.

One year prior to this visit, she had visited the provincial hospital due to abdominal distension. A computerized tomography (CT) scan of the abdominopelvic region revealed a large amount of ascites with peritoneal nodule and pleural effusion. Abdominal paracentesis and pleural tapping were performed, which indicated exudative fluid, but no malignant cells were noted. She was then referred to another hospital for further work-up. Another CT scan of the abdominopelvic region was performed, which revealed circumferential wall thickening at the mid to upper rectum suggesting rectal cancer with a large amount of ascites and right pleural effusion. Colonoscopy and tissue biopsy at the rectum showed only acute colitis with no dysplasia or malignancy detected. However, she did not return to follow up after this operation.

**Figure 1:**
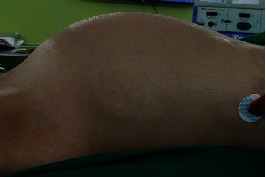
Physical examination revealed a markedly distended abdomen secondary to a large amount of ascites.

**Figure 2:**
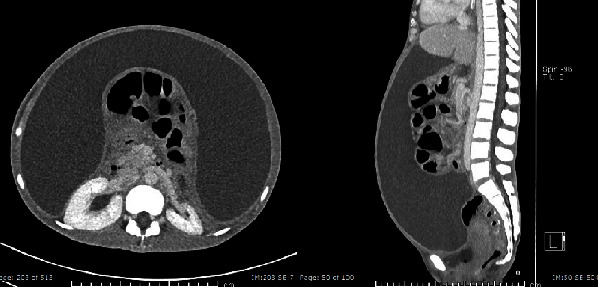
Computed tomography of the abdominopelvic region shows massive loculated ascites with internal thin septation, causing posterior displacement of the intraperitoneal organs.

Upon presentation at our hospital, the patient appeared chronically ill and emaciated with a body mass index of 14.9 kg/m^2^. Her abdomen was markedly distended due to a large amount of ascites (Figure 1). There were no masses or pain upon palpation. Vaginal examination revealed bulging of the anterior vaginal wall due to ascites with no palpated pelvic mass. A CT scan of the abdominopelvic region performed at our hospital showed infiltrative enhancing lesions involving the cul-de-sac and perirectal region with massive loculated ascites and internal septation. The uterus and both ovaries appeared unremarkable (Figure 2). A chest X-ray revealed bilateral pleural effusion and passive atelectasis of both lower lobes. Her CA-125 level was 112 IU/mL, CA19-9, and CEA levels were within the normal range.

**Figure 3:**
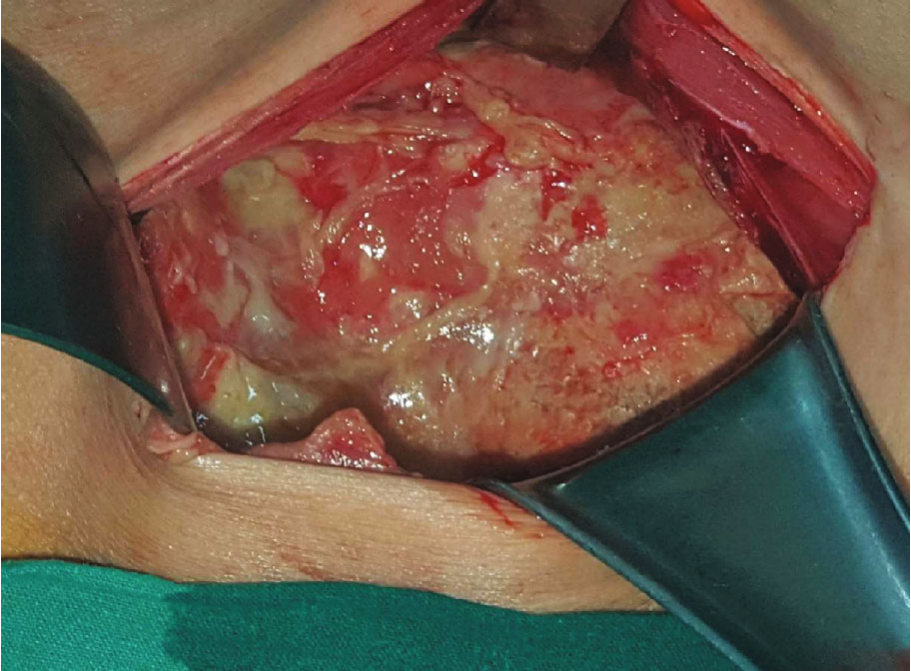
Intraoperative findings revealed thickened matted bowel loops and necrotic debris covering the entire peritoneal surface and viscera.

We performed abdominal paracentesis, which yielded a clear yellowish fluid. Cytologic examination suggested inflammation without malignant cells. The ascites sample was also sent for tuberculosis PCR and acid-fast bacilli staining, both of which were negative. Exploratory laparotomy was scheduled for tissue diagnosis. Upon laparotomy, we noted a large amount of serosanguinous ascites, thickened matted bowel loops, and necrotic debris covering the entire peritoneal surface (Figure 3). The right adnexa and uterus were partially identified. No gross focal mass lesions were found. The surgical procedures included drainage of 6.5 liters of ascites, lysis adhesion, biopsy of the peritoneum, and right salpingo-oophorectomy.

**Figure 4:**
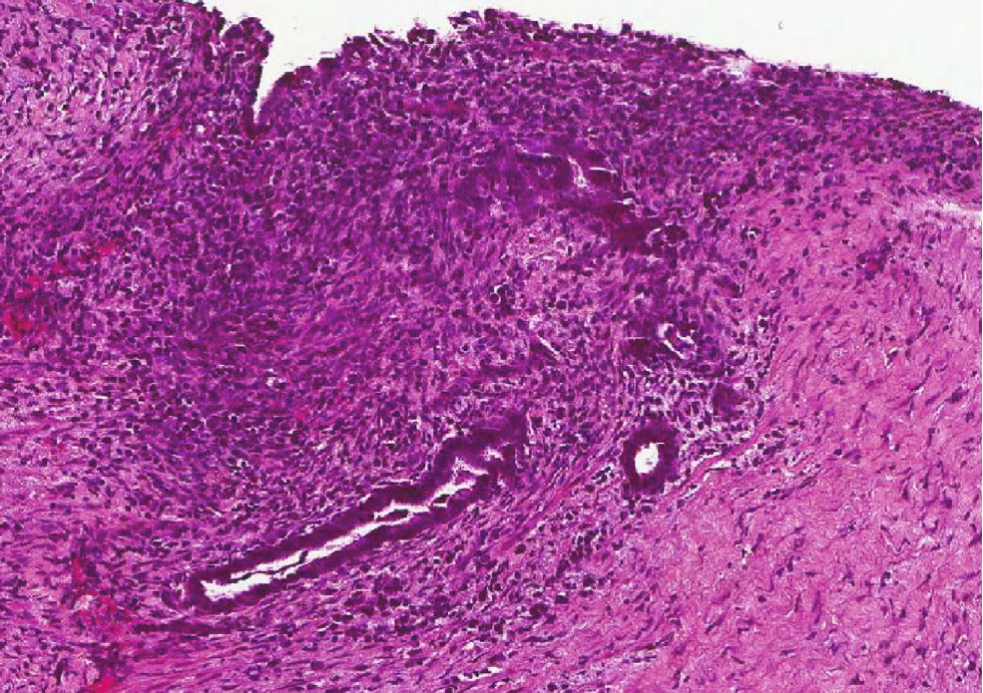
Microscopic examination of the peritoneum revealed benign endometrial glands with stroma in a thick fibrous tissue with background of chronic inflammation (H&E staining; 10x).

**Figure 5:**
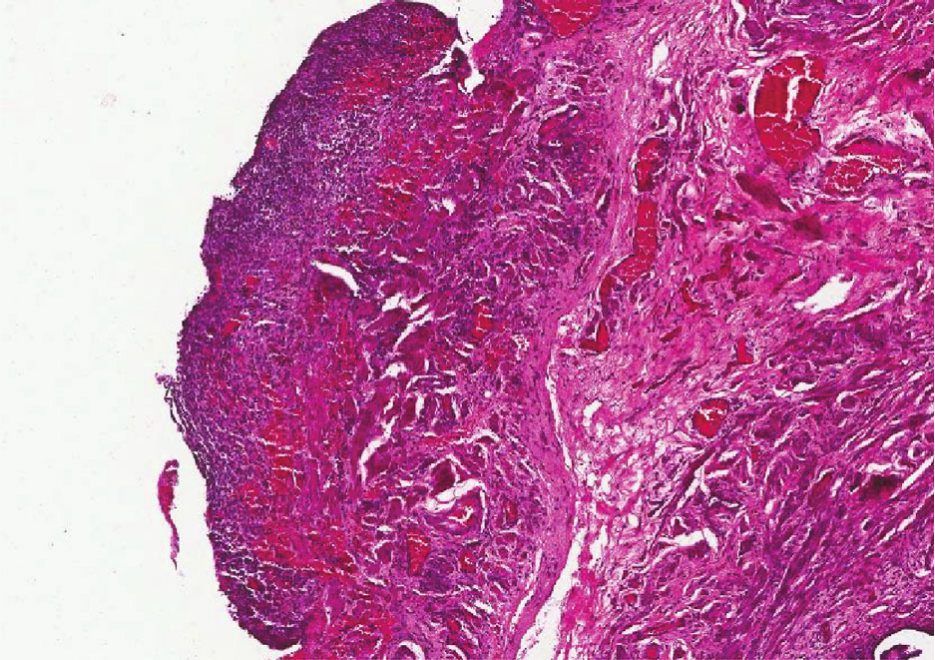
Microscopic examination of broad ligament adjacent to the right fallopian tube revealed endometriotic foci (H&E staining; 10x).

Histologic examination of peritoneum tissue showed benign endometrial glands with stroma deposited within a thick fibrous tissue and chronic inflammation background (Figure 4). Immunohistochemical studies of peritoneal tissue showed positive staining for estrogen receptor (ER) and progesterone receptor (PR) within the foci of endometriosis. Endometriotic foci at the broad ligament were found adjacent to an unremarkable right fallopian tube (Figure 5). Sections of the right ovary were unremarkable. There were no malignant cells in any of the tissues examined. Cytologic examination of ascites fluid revealed inflammation without malignant cells. A final diagnosis of endometriosis was made based on these pathological and cytological findings.

The patient's postoperative course was uneventful. GnRH analog was prescribed for six months postoperatively. The patient has subsequently been on a daily regimen of oral progestin. At fifteen months following the operation, the patient had no symptoms and the results of a physical examination were unremarkable.

## 3. Discussion

Although endometriosis has been implicated as a precursor for certain types of epithelial ovarian cancer [7, 8], the occurrence of ascites secondary to endometriosis is rarely encountered, particularly among Asian women. This entity simulates gynecological malignancy and is seldom recognized before surgical exploration of the abdomen. The occurrence of this condition in a multiparous Asian woman is unique in the case literature.

The term ascites describes the pathologic accumulation of fluid within the peritoneal cavity [9]. Cirrhosis of the liver is the most common cause of ascites, but other conditions, such as heart failure, kidney failure, infection (such as tuberculosis), cancer, or gynecological malignancy can also cause this condition [10]. When a woman presents with ascites, exclusion of certain gynecological disorders, including ovarian cancer, primary peritoneal cancer, fallopian tube cancer, and pelvic tuberculosis, is necessary. Ascites secondary to endometriosis is a rare phenomenon, of which the first case was reported in 1954 [11] and only approximately 60 cases have been reported worldwide [6, 12]. The clinical presentation in our patient is generally consistent with those that have previously been reported, except with regard to the patient's parity status and ethnicity. Reported cases have mostly been in African (82%) and nulliparous (85%) women [13]. The main presenting symptoms in these cases were abdominal distention, pain, and/or weight loss [6, 12–18]. Significant weight loss occurred in one-third of reported cases, and concurrent pleural effusion was found in 38% of cases [6]. Additionally, as in our report, the patient usually had a history of endometriosis-related symptoms including dysmenorrhea and pelvic pain.

Owing to the rarity of massive ascites associated with endometriosis, the diagnosis of this clinical entity is made after the exclusion of other more common causes. The presentation of ascites, weight loss, and peritoneal lesions mimic advanced gynecologic malignancy and peritoneal tuberculosis. Consequentially, most of the cases reported, including that in this report, underwent abdominal paracentesis to obtain an ascites fluid sample for cytological examination in order to exclude malignant cells and tuberculosis infection. The appearance of ascites fluid secondary to endometriosis could be bloody, dark brown, or serosanguineous [6, 12, 13]. Serum CA-125 levels were also investigated in most reported cases and varied from 20 to 5,000 IU/mL [12]. In our case, the patient's CA-125 levels were slightly elevated. Although an associated cancer or other diseases were not found and were not likely, long-term surveillance is required to reaffirm the final diagnosis.

The definitive diagnosis of endometriosis with ascites is made upon the operative assessment and histological examination of a biopsied tissue. Extensive adhesions have often been encountered upon operation. Extrapelvic endometriosis has usually involved surrounding structures including the omentum, bowel, or pelvic organs [17]. Intraoperative findings in our case were similar to those previously reported. A definite diagnosis was made upon histological confirmation of endometrial tissue in the peritoneum and board ligament.

The pathogenesis of endometriosis-associated massive ascites remains unknown, so there is yet no specific treatment for this rare clinical entity [5, 19, 20]. Since endometriosis is estrogen-dependent, suppression of ovarian function through surgery and/or medication is necessary to prevent recurrence. Surgical management can be either conservative or radical depending on patients' age, the severity of the disease, and the desire for fertility. If feasible, radical surgery may be the treatment of choice for women who present with ascites. Long-term medical treatment is required following conservative treatment [5].

Due to the fact that almost all patients with endometriosis-associated massive ascites have been of reproductive age and nulliparous, conservative surgery and postoperative medication has been the most common treatment protocol. Medications for suppressing ovarian function include GnRH agonist, progestogenic agents, and combined hormonal contraception. Although our patient was multiparous, radical surgery could not be carried out due to extensive adhesion. Hence, ascites drainage, lysis adhesion, biopsy of the peritoneum, and right salpingo-oophorectomy were performed. Postoperative GnRH agonist was prescribed for six months followed by long-term administration of a progestogenic agent. During the treatment, there was no evidence of ascites reaccumulation.

## 4. Conclusion

Herein, we describe a case of endometriosis diagnosed in a reproductive-aged Asian woman who presented with massive ascites. Evidence to support the diagnosis of endometriosis-associated massive ascites includes the two-year history of ascites accumulation, exclusion of other possible causes, and response to hormonal suppression. The clinical course of our case was similar to those previously reported in patients of other ethnicities. This suggests that, despite its extreme rarity, clinicians should include endometriosis in the differential diagnoses of Asian women presenting with massive ascites.
